# Sex-Specific Risk of Smoking for Abdominal Aortic Aneurysm and Exploration of Potential Mechanism: Meta-Analysis and Prospective Cohort Study

**DOI:** 10.1161/ATVBAHA.125.322601

**Published:** 2025-05-08

**Authors:** Paul Welsh, Anna Louise Pouncey, Naveed Sattar, Janet T. Powell

**Affiliations:** 1 School of Cardiovascular and Metabolic Health, University of Glasgow, United Kingdom (P.W., N.S.).; 2 Department of Surgery and Cancer, Imperial College London, United Kingdom (A.-L.P., J.T.P.).

**Keywords:** aortic aneurysm, abdominal, inflammation, risk, sex, smoking

## Abstract

**BACKGROUND:**

Smoking is the strongest modifiable risk factor for the development of abdominal aortic aneurysm (AAA). This study aims to confirm whether smoking is a stronger risk factor in women than men and identify contributory reasons, including inflammation, for any sex-specific difference observed.

**METHODS:**

Systematic review and meta-analysis, conducted according to Preferred Reporting Items for Systematic Reviews and Meta-Analyses guidance. Data sources were Medline, Embase, and Cochrane Central Register of Controlled Trials. Population-based studies reporting the risk of AAA, adjusted for age and cardiovascular risk factors, for women versus men, were included. These were complemented by data from UK Biobank, which also were assessed for sex-specific effects of smoking on incident atherosclerotic cardiovascular disease.

**RESULTS:**

Meta-analysis of 6 studies (including UK Biobank, 2001–2024) showed that the relative risk ratio of current versus never smokers for incident AAA in women versus men was 1.78 (95% CI, 1.32–2.38). In the UK Biobank cohort, the sex-specific relative risks of current smoking risks were higher for AAA than for atherosclerotic cardiovascular disease; hazard ratio, 1.74 and 1.29, respectively: analysis by cigarettes per day echoed these findings, but pack-year history had lesser association. The proportionately lower AAA incidence rate in exsmokers (versus current smokers) was more marked in women. Sex-specific risks of current smoking for incident AAA were not significantly modified by CRP (C-reactive protein), white blood cell count, or other inflammatory markers.

**CONCLUSIONS:**

The relative risk of developing AAA by current smokers is almost twice as high in women versus men, but no strong association with inflammation was observed. Other reasons, including smoking intensity, must be sought.

**REGISTRATION:**

URL: https://www.crd.york.ac.uk/PROSPERO/; Unique identifier: CRD2024586609.

**Highlights**

- Current smoking is associated with a ≈1.8-fold higher relative risk of developing an abdominal aortic aneurysm in women compared with men.
- The proportionately lower abdominal aortic aneurysm incidence rate in exsmokers (versus current smokers) was more marked in women.
- Sex-specific risks of current smoking for incident abdominal aortic aneurysm were not significantly modified by CRP (C-reactive protein), white blood cell count, or other inflammatory markers.
- In the UK Biobank cohort, the sex-specific relative risks of current smoking risks were higher for abdominal aortic aneurysm than for atherosclerotic cardiovascular disease.

Smoking is a stronger risk factor for abdominal aortic aneurysm (AAA) than for the incidence of most other common forms of cardiovascular disease.^1^ Three studies, 2 longitudinal and 1 screening, have previously indicated the relative effect of smoking on AAA development appears to be stronger in women than men.^2–4^ Because AAA is up to 6× more common in men^5,6^ and recently smoking rates among adult men are only slightly higher than in women,^7^ such observations may seem surprising. However, it is recognized that men and women may smoke differently and for different reasons.^8,9^ Men and women choose different types of cigarettes^10^ and appear to inhale cigarettes differently.^8^ Neuroimaging studies support the activation of differential brain pathways, with men smoking primarily for the reinforcing or reward effects of nicotine, whereas women tend to smoke to regulate mood or in response to cigarette-related cues.^9^

The culmination of cultural and physiological differences may result in different health outcomes. There is strong evidence that the risks of smoking for the development of both coronary heart disease and chronic obstructive pulmonary disease are stronger in women than men.^11–13^ No underlying mechanisms have been identified. The strength of the association of smoking with the development and growth of AAA may offer a better possibility of identifying mechanisms.^1,14^ Therefore, the aim of this study is to consolidate the evidence for sex-specific differences in AAA through systematic review and meta-analysis and explore UK Biobank data for underlying mechanisms. Because inflammation is important in the pathogenesis of chronic obstructive pulmonary disease, and may be relevant to coronary heart disease and AAA, the hypothesis that smoking induced a higher inflammatory response in women than in men, which mediated the sex-specific effect on AAA risk, was explored.

## Materials and Methods

The data that support the findings of this study are available from the corresponding author upon reasonable request.

### Systematic Review

The systematic review was registered at https://www.crd.york.ac.uk/PROSPERO/ (unique identifier: CRD2024586609) and was conducted and is reported using the Preferred Reporting Items for Systematic Reviews and Meta-Analyses statement (https://www.prisma-statement.org/; Figure S1). Using ProQuest Dialog, a search strategy for MEDLINE, EMBASE, and Cochrane Central Register of Controlled Trials using a combination of controlled vocabulary (MeSH terms and free text terms) was formulated with the initial search conducted on August 31, 2024, with searches updated including the additional MESH term gender on December 31, 2024. The initial MeSH terms used were smoking or cigarettes or tobacco and sex and abdominal aortic aneurysm, with free text terms of pack-years, population, screening, and longitudinal studies. There were no date restrictions. Only publications that included an English language abstract were included, and the reference lists of any reviews or systematics reviews identified were searched manually. If necessary, study authors were contacted for further information. The essential inclusion criterion was that the risk of AAA was compared for current smokers and never smokers for men and women separately. Other inclusion criteria were population-based studies, risk results presented with 95% CIs, and either adjusted at least for age or providing data to enable age adjustment. Eligible studies could provide adjusted odds ratios, hazard ratios (HRs), or relative risks (RRs) and their 95% CI or provide data to enable estimation of such a risk. Risk of bias was assessed using the ROBINS-E tool (https://www.riskofbias.info/welcome/robins-e-tool; Figure S2). Odds ratios and HRs (assuming constant hazards over time) were converted to RRs using the baseline risk or the probability of the event in the control group and the delta method for approximation of the variance. Exclusion criteria included reports only in languages other than English, reports of hospital studies or AAA repairs, and studies with low (<10%) representation of either sex and case-control studies with <200 AAA cases. Data were extracted by 2 independent reviewers onto Excel spreadsheets. Meta-analysis of RR for men and women, and of RR ratios for women to men, was conducted using the inverse variance method with the DerSimonian-Laird estimator for τ^2^, the Jackson method for the CI of τ^2^, and Hartung-Knapp adjustments for random effects, with graphical representation using forest plots. A *P* value of <0.05 was considered significant. The Forest plots also show the prediction interval, which is the range in which the point estimates of 95% of future studies are expected to fall (assuming that the effect sizes are normally distributed). Heterogeneity was assessed using a χ^2^ test and the I^2^ statistic. Further leave-one-out and influence analyses, using Baujat plots, externally standardized residuals, DFFITS (difference in fit statistic) value, Cook distance, covariance ratio, leave-one-out τ^2^, and Cochran Q values to identify studies to exclude, were used to examine the robustness of findings. Analyses were conducted in R Studio (https://www.rstudio.com) using the DMetar package.^15^

### UK Biobank Data and Cohort Selection

The original data used in this study are available via UK Biobank (https://www.ukbiobank.ac.uk/), subject to necessary approvals. UK Biobank is a large population-based cohort study of 502 188 participants ranging in age from 37 to 73 years, recruited between 2006 and 2010.^16,17^ All participants underwent an assessment at 1 of 22 centers across England, Scotland, and Wales, where touch-screen questionnaires recorded health and lifestyle information, participants underwent an interview for health conditions and medication use, and a wide range of biological measurements were taken. Covariates in the analysis included age, sex, ethnicity (White, Black, South Asian, and other), systolic and diastolic blood pressures (BPs; both the average of 2 measurements), Townsend deprivation score (a postcode-based measure of socioeconomic deprivation), body mass index, weight, height, body fat percentage from bioimpedance, diabetes (type 1 or 2), IPAQ (International Physical Activity Questionnaire)–measured physical activity (low, moderate, or high), chronic kidney disease, baseline atherosclerotic cardiovascular disease (ASCVD), and lung function (forced expiratory volume in 1 s and forced vital capacity). Medications included BP-lowering medications, cholesterol-lowering medications, and antiplatelet medications obtained for self-report or nurse interview. Blood biomarkers were obtained according to a standardized protocol.

The primary exposure of interest was smoking status, categorized as never, exsmoker, and current smoker. Smoking habit was also explored using the number of cigarettes smoked per day (zero in nonsmokers, imputing unknown smokers as the median per day, and 10 cigarettes a day among pipe and cigar smokers) and using pack-years of smoking (UK Biobank data field 20161). These were analyzed to assess whether the sex interaction held after accounting for smoking intensity and cumulative exposure.

Participants were excluded if they self-reported aortic aneurysm, aortic aneurysm rupture, or aortic dissection (UK Biobank data field 20002) or first occurrence of aortic aneurysm or aortic dissection was recorded as occurring before recruitment (UK Biobank data field 131382; total n excluded=591). We conducted a complete case analysis in 382 762 participants with complete data for the primary adjustment model.

UK Biobank received ethical approval from the North West Multi-Centre Research Ethics Committee (reference: 11/NW/03820). All participants gave written informed consent before enrollment, in accordance with the principles of the Declaration of Helsinki. This project was performed under UK Biobank (project approval No. 71392).

### Outcomes

All clinical data in the hospital inpatient data were coded according to the World Health Organization’s *International Classification of Diseases, Tenth Revision*, codes. All operations and procedures were coded according to the Office of Population, Censuses and Surveys: Classification of Interventions and Procedures codes. Dates and causes of death were obtained from death certificates held by the National Health Service Information Centre for participants from England and Wales and the National Health Service Central Register Scotland for participants from Scotland.

Implementation of the United Kingdom National Health Service screening policy for AAA in men aged ≥65 years was complete in most parts of the United Kingdom by late 2009.^17^ Participants with complete data for primary adjustment variables (vide infra) were followed up until August 31, 2022, in Scotland, October 31, 2022, in England, and May 31, 2022, in Wales. The outcome of interest, incident AAA, was defined from first hospital inpatient report of AAA or death from AAA (both based on the *International Classification of Diseases, Tenth Revision*, codes I71.3 and I71.4) or an AAA-related surgical procedure_._^18^ A secondary outcome was ASCVD, defined as death from coronary heart disease or stroke (I20-I25 and I60-I64), or hospitalization for myocardial infarction or stroke (I21-I22 and I60-I64). The partial cohort coverage data from general practitioner registers were not used to ascertain either AAA or ASCVD.

### Statistical Methods for UK Biobank Data

Rate of AAA was obtained from crude estimates per 10 000 person-years stratified by smoking status and sex. Descriptive statistics were generated for baseline characteristics, stratified by AAA and ASCVD outcomes, using means, SDs, and *t* tests for normally distributed continuous variables; medians, and interquartile intervals, and rank-sum tests for skewed variables; and counts, percentages, and χ^2^ tests for categorical variables.

Cox proportional hazards regression models were used to evaluate the association between smoking status, sex, and outcomes, with AAA and ASCVD. Interaction terms between sex and smoking-related variables were included in the models to assess potential effect modification. Models were adjusted for the following confounders: age, ethnicity, Townsend deprivation score, diabetes status, total cholesterol, HDL cholesterol, BP medication use, statin use, baseline ASCVD, and chronic kidney disease. Subsequently, there were 2 sequential sensitivity analyses adjusting for other potential variables, which might modify the sex-specific effect identified. These included systolic BP, diastolic BP, body mass index, body mass index interaction with diabetes status, body fat percentage, HbA1c (glycated hemoglobin), markers of inflammation (ALP [alkaline phosphatase], ALT [alanine aminotransferase], AST [aspartate aminotransferase], GGT [γ-glutamyltransferase], CRP [C-reactive protein], and white blood cell [WBC] count), and factors associated with thrombosis (platelet count and antiplatelet medication use). Second, an extended mediation model additionally included neutrophil-to-lymphocyte ratio, lung function (forced expiratory volume in 1 s and forced vital capacity), and physical activity (IPAQ), with sex interactions for forced expiratory volume in 1 s and forced vital capacity. This latter extended model had a lower sample size due to missing data on lung function and physical activity. Analyses by number of cigarettes smoked per day and pack-year history were used to further investigate smoking habits. Stratified analyses by CRP concentration and WBC were performed to further assess the role of inflammation. All analyses were for complete cases and conducted using Stata, version 18 (StataCorp, College Station, TX). For AAA, a sensitivity analysis was conducted evaluating the effect of current smoking above and below the median baseline age of those with hospital admissions or death from AAA (incident AAA).

## Results

### Systematic Review and Meta-Analysis

Systematic review of the literature, to December 31, 2024, identified 466 potential studies, which after screening of the title and abstract left 31 potential studies. This reduced to 5 reports after review of the full article and data extraction.^2–4,18,19^ In addition, a search was conducted for UK Biobank studies for potentially available information, yielding 1 further study.^20^ Many of the 31 potential studies were excluded because they either did not report sex-specific analyses or did not provide separate results for exsmokers and current smokers. Additional data were requested from the 2024 Copenhagen study, but there was insufficient power due to few women with AAA.^5^ The Preferred Reporting Items for Systematic Reviews and Meta-Analyses flowchart and risk-of-bias assessment are shown in Figures S1 and S2, and details of the included studies are provided in Table 1, with a total of 1 050 810 men and 1 771 349 women. Overall, studies had a low-medium risk of bias.

**Table 1.**
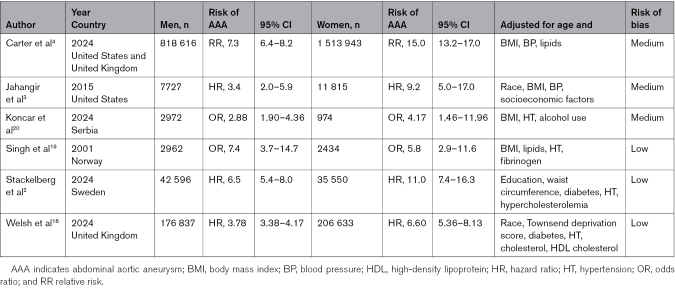
Studies Included in the Meta-Analysis or Risk of AAA in Current Smokers Versus Never Smokers

**Figure 1.**
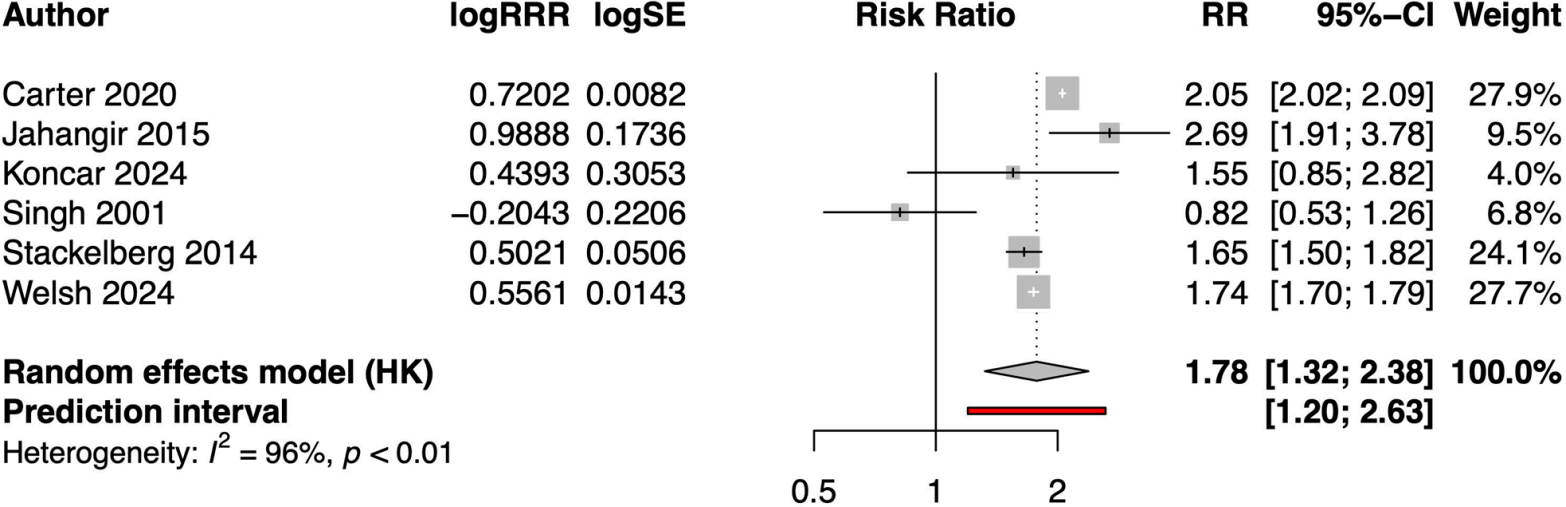
**Forest plot of relative risk ratios (RRRs; women to men) of risk of abdominal aortic aneurysm in current vs never smokers.** HK indicates Hartung-Knapp adjustments for random effects; and RR, relative risk.

For men, the pooled RR of AAA in current smokers versus never smokers was 4.83 (95% CI, 3.0–7.62), with high heterogeneity (I^2^=94%). For women, the pooled RR of AAA of current smokers versus never smokers was 8.44 (95% CI, 5.25–13.92), also with high heterogeneity (I^2^=90%). The pooled RR ratio, women to men, was 1.78 (95% CI, 1.32–2.38), again with high heterogeneity (I^2^=96.2%) and is shown in Figure 1. All findings were robust on leave-one-out in influence analyses (Figures S3 and S4). Overall, these data consistently indicate that the RR for development of AAA associated with current smoking is almost twice as high in women as in men.

### Comparison of the Sex-Specific Risks of Smoking Associated With Incident AAA and ASCVD in the UK Biobank Cohort

**Figure 2.**
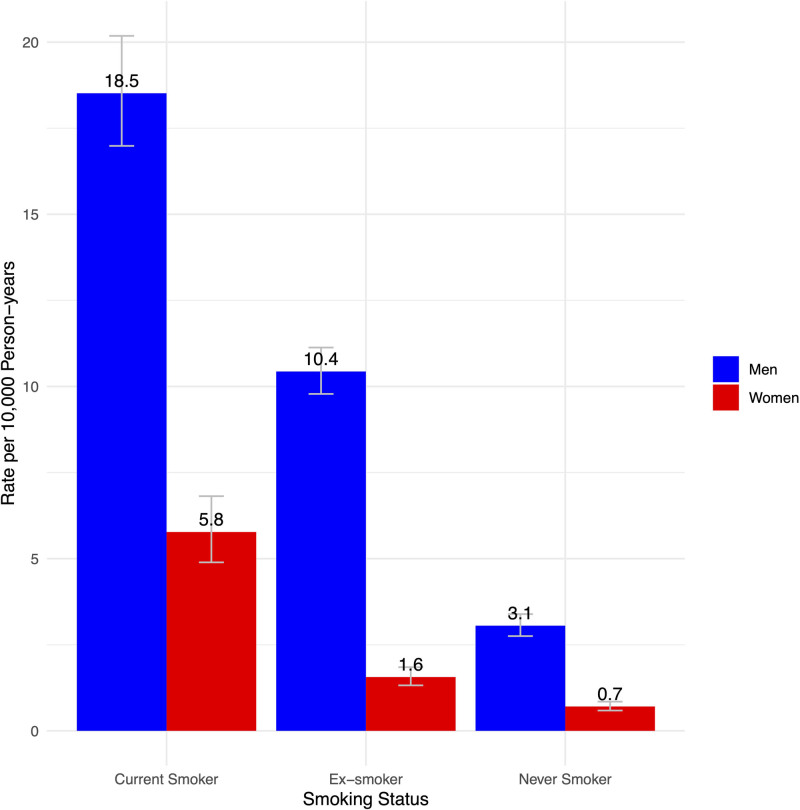
Rates of incidence of abdominal aortic aneurysm in the UK Biobank cohort by smoking category for men and women.

The population included 176 508 men and 206 254 women, with incident AAA diagnosed in 1793 and 393, respectively, with baseline characteristics shown in Table 2. Women had lower pack-year histories of smoking than men. Other cardiovascular risk factors also differed by sex, with higher use of prevention medications in men than women. Men also had higher BPs, while women had higher serum cholesterol and CRP concentrations. Over a median 13.4 years of follow-up (IQR, 12.9–14.3) the rate of AAA per 10 000 person-years was 7.7 (95% CI, 7.4–8.1) in men and 1.4 (95% CI, 1.3–1.6) in women. The unadjusted rates for AAA incidence per 10 000 person-years in men and women in never smokers, exsmokers, and current smokers are shown in Figure 2; rates of incident AAA were higher in smokers in both sexes, but the absolute risk of AAA was always higher in men regardless of the smoking status. The proportionately lower AAA incidence rate observed for exsmokers compared with current smokers appears to be more marked in women than in men (72% lower for women versus 44% lower for men; Figure 2).

**Table 2.**
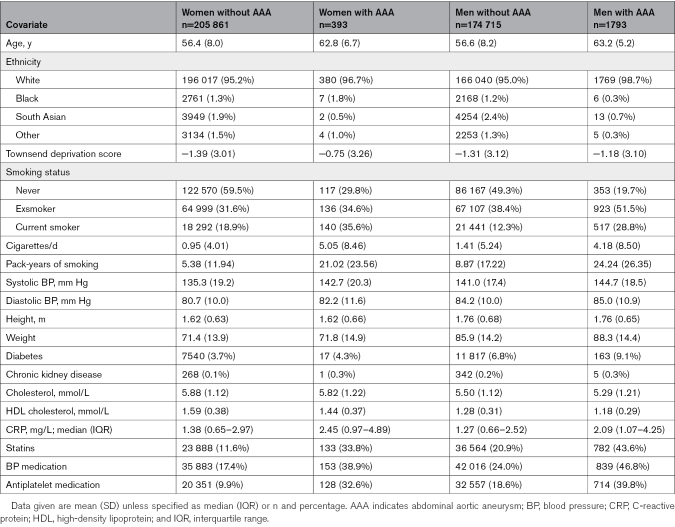
Baseline Characteristics of Women and Men by Incident AAA Status During Follow-Up in the UK Biobank Cohort

**Figure 3.**
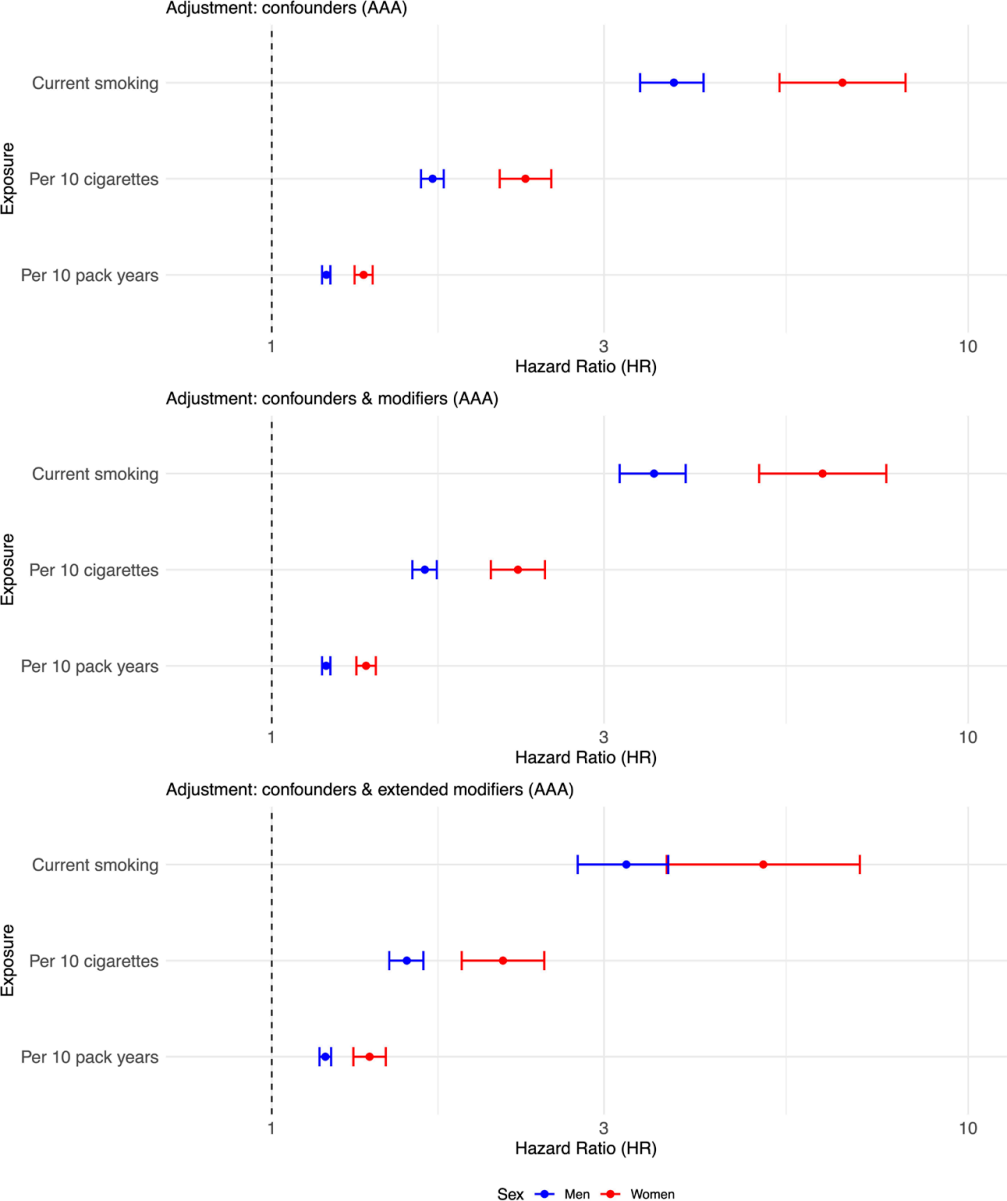
**Forest plot showing the similar sex-specific differences in risk of abdominal aortic aneurysm (AAA) in current vs never smokers from UK Biobank for analysis by number of cigarettes smoked per day (in multiples of 10) and by pack-year history (in multiples of 10).** All data were adjusted for age, ethnicity, Townsend deprivation score, diabetes status, total cholesterol, HDL (high-density lipoprotein) cholesterol, blood pressure medication use, statin use, baseline atherosclerotic cardiovascular disease, and chronic kidney disease. The modifiers and extended modifiers are described in Statistical Methods for UK Biobank Data. HR indicates hazard ratio.

The primary adjusted HRs for current smokers versus never smokers in men and women for incident AAA are 3.78 and 6.60, respectively (Figure 3, top). The test for sex interaction was highly significant (HR, 0.57 [95% CI, 0.45–0.72]). The data used in these analyses are shown in Table 2.

**Figure 4.**
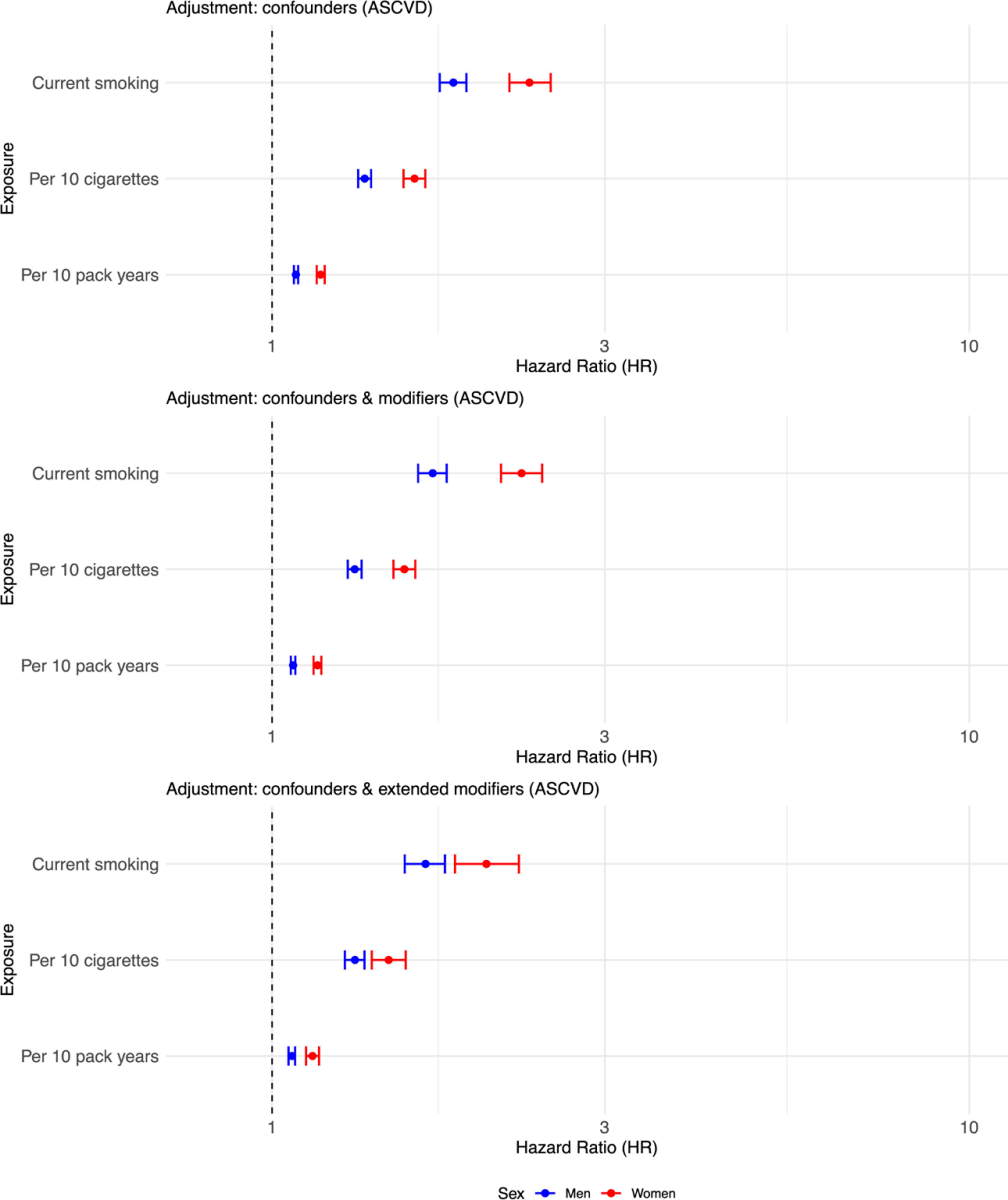
**Forest plot showing the similar sex-specific differences in risk of atherosclerotic cardiovascular disease (ASCVD) in current vs never smokers from UK Biobank for analysis by number of cigarettes smoked per day (in multiples of 10) and by pack-year history (in multiples of 10).** All data were adjusted for age, ethnicity, Townsend deprivation score, diabetes status, total cholesterol, HDL (high-density lipoprotein) cholesterol, blood pressure medication use, statin use, and chronic kidney disease. The modifiers and extended modifiers are described in Statistical Methods for UK Biobank Data. HR indicates hazard ratio.

The baseline demographic data for the ASCVD cohort are given in Table S2. The comparable HRs for incident ASCVD in men and women, for the same cohort, in current smokers versus never smokers were 1.82 (95% CI, 1.74–1.90) and 2.34 (95% CI, 2.19–2.51), respectively (Figure 4; Table S3). The test for sex interaction was highly significant but weaker than for AAA (HR, 0.78 [95% CI, 0.72–0.84]).

### Investigation of Potential Modifiers of the Sex-Specific Effects of Smoking for Incidence of AAA in the UK Biobank Cohort

Neither of the sequentially adjusted comparisons of the risk of AAA in current smokers versus never smokers showed an important amelioration of the sex-specific difference in primary adjusted HRs, although the lower numbers in the second sequential analysis led to much wider CIs (Figure 3, top). The analyses included the covariate markers of inflammation: CRP, alkaline phosphatase, and WBC, with neutrophil/monocyte ratio included in the extended modifiers; details of all the additional covariates are shown in Table S1.

Next, we considered the effects of the intensity and extent of smoking habits. At baseline, women with incident AAA smoked more cigarettes per day than men with incident AAA, but pack-year history was higher in men (Table 2). The forest plots show the association of the number of cigarettes smoked per day and pack-year smoking history with AAA (Figure 3, middle and bottom, respectively). The number of cigarettes per day showing a stronger sex-specific difference than pack-year smoking history: test for sex interaction in the primary adjusted analyses: HRs, 0.736 (95% CI, 0.671–0.806) and 0.884 (95% CI, 0.856–0.913) respectively. For comparison, the association of smoking habit with the sex-specific RRs of ASCVD is shown in Figure 4: the effects of the number of cigarettes per day also appeared stronger than pack-year history (Figure 4): test for sex interaction in the primary adjusted analyses: 0.848 (95% CI, 0.814–0.884) and 0.922 (95% CI, 0.908–0.935), respectively. A comparison of splines that allow for nonlinearity confirmed higher RRs of current smoking for AAA in women compared with men (Figures S5 and S6). For both number of cigarettes per day and pack-years, the HR for AAA increased more steeply in women. Increasing exposure to >30 pack-years or smoking >15 cigarettes/d had the greatest effect on increasing the risk of AAA in women.

To further investigate the role of inflammation, analyses stratified by CRP concentrations <2 and ≥2 mg/L indicated a weak potentiation of the HR for sex interaction in the upper CRP stratum (Table 3). For WBC, the converse was observed: the HR for sex interaction was weaker in the upper WBC stratum (Table 3).

**Table 3.**
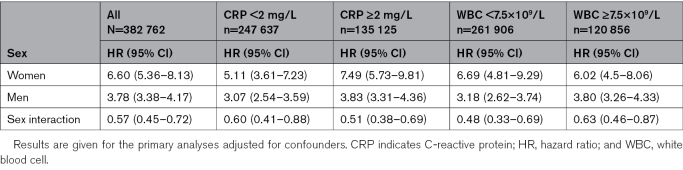
The Risk of Current Versus Never Smoking on Incident Abdominal Aortic Aneurysm Stratified by CRP Concentration and WBC

Because AAA is a disease of aging, a sensitivity analysis was conducted comparing the sex-specific effect of smoking stratified by median baseline age of those with incident AAA (64 years). The test for sex interaction in the primary adjusted analyses was similar for those <64 years at baseline and those aged ≥64 years at baseline; HR, 0.62 (95% CI, 0.44–0.88) and 0.52 (95% CI, 0.38–0.72), respectively, with little change after adjustment for modifiers (Table S4).

## Discussion

The meta-analysis consolidates and extends the evidence from previous reports, putting improved precision on the RR of AAA in current smokers, which is almost twice as high in women versus men. The sex-specific RR ratio of smoking for AAA was much higher (1.78-fold) than the more modest 1.25-fold increase for coronary heart disease observed previously^11^ or for ASCVD in the present UK Biobank cohort (RR ratio, 1.29). This study is the first to explore potential modifiers that might help explain mechanisms underpinning the higher RR of smoking in women versus men. There was only the weakest of evidence to support our hypothesis that this was mediated by inflammation, or any other covariate investigated, and the sex interaction was still notable by smoking history, particularly the number of cigarettes smoked per day. The rate of AAA incidence was low in female nonsmokers (0.7 per 10 000 patient-years) in the UK Biobank cohort. A low incidence in nonsmokers is likely to contribute to the high RR associated with smoking in women, since AAA is rare in women who have never smoked. Other possible contributory reasons for the high RR of AAA in female smokers include the much lower use of cardiovascular risk prevention medications in women or that men who have never smoked are at higher risk of AAA due to their lower levels of non-HDL cholesterol and higher BPs.

The RR of current smoking for AAA in women varied from 0.82 to 2.69 in the different studies; pooled RR of 1.78 (95% CI, 1.32–2.38), with high heterogeneity. Reasons for the high heterogeneity observed might be related to underlying population and ethnic differences, the varying study designs (cross-sectional or longitudinal), the mode of AAA ascertainment (screening in some studies for point prevalence or record-linkage clinical incidence in others), or even the definition of AAA (aortic diameter either 3.0 or 3.5 cm in screening studies with various planes of measurement). In the UK Biobank cohort, some of the AAA in men could have been detected through the national screening program, which is for men only. However, the majority of screen-detected AAAs are small, in the early phases of development.^21^ The small AAAs will not have been included in our UK Biobank cohort of clinically relevant AAA, which is based on hospital admissions and deaths. Women are not routinely offered screening. Although the thresholds for both diagnosis (3 cm) and intervention (5.5 cm) are the same as for men, women have smaller aortic diameters.^22,23^ Therefore, both incident and screen-detected AAAs in women could have reached a more advanced stage of their clinical course than in men. This could accentuate the sex disparity associated with current smoking noted for AAA. The increased risks of current smoking for the development of AAA in women might be offset by their proportionately lower rate of AAA incidence from stopping smoking, as suggested by the UK Biobank data in Figure 2.

Inflammation is an important pathogenic factor in other disorders where the sex-specific risks of smoking have been described, as it is for AAA. Our first hypothesis for the high excess risk of smoking for AAA in women was that it was mediated by inflammation. Indeed, baseline CRP concentrations were higher in women than in men, but including CRP as a covariate in regression models or stratifying analyses by baseline CRP concentration had minimal effect on the sex-specific interaction. These analyses indicated that increasing CRP concentration, as a reflection of low-grade inflammation, had only a weak effect on potentiating the RR of smoking in women versus men, while analyses stratified by baseline WBC showed a weak effect in the opposite direction.

Further analysis of smoking in the UK Biobank cohort demonstrated a similar relative sex-specific disparity in risk of AAA by the number of cigarettes per day (higher in women) but slightly less disparity for total smoking exposure (pack-years, which was lower in women). A similar trend was observed for ASCVD. This could suggest that smoking more cigarettes per day might contribute to the high risk of current smoking for AAA in women. Previously, a small case-control study of smokers with AAA compared with smokers with occlusive aortic atherosclerosis identified depth of inhalation as a potential risk factor for AAA,^24^ and an increasing amount of daily smoking also has been associated with increased risk of AAA in men.^25^ There is ample evidence of genetic risk factors for smoking, as well as evidence of sex-specific differences in the neural processing of cigarette smoking.^26^ Therefore, a fruitful approach to understanding the mechanisms underlying the sex-specific effects of smoking in cardiovascular disease might be genetic investigation of the nicotine signaling pathways in the brain. This is an area where sex-specific differences in dopamine signaling pathways are increasingly important in neuropsychiatric disorders, including addiction, with sex-specific differences in dopamine receptor distribution, density, and function.^27^

Sex-specific endocrine interactions are another potential contributor to the sex-specific differences in the risk of smoking for cardiovascular disease. There is a dose-dependent effect of pack-year history of smoking on risk of early menopause, which is likely to accelerate vascular aging in women.^28^ The mechanisms underlying this effect on early menopause are various, but direct toxicity of smoking on ovarian follicles is supported by the dose-dependent effect of smoking (number of cigarettes smoked) to reduce Mullerian hormone concentrations.^29^

Numerous biological pathways have been associated with AAA development spanning molecular damage to the aorta from the luminal thrombus to medial degeneration to neovascularization and immune cell infiltration in the adventitia. Many of the specific cell and molecular pathways have been elucidated in animal models. Some of these models incorporate cigarette smoke exposure to mimic the human disease.^30^ The data presented here reinforce the need to use both male and female animal models and analyze their data separately.

There are several limitations to our study. Only 6 studies contributed to the meta-analysis, too few for examining the risk of publication bias. Three studies, including UK Biobank, were longitudinal but only used baseline data for their analyses of smoking habits.^2,3,18^ None of these longitudinal studies made allowance for the competing risk of death from other causes. It is important to recognize that UK Biobank reports on a healthier-than-average population and in which there may be many unconsidered or unmeasured confounders.^31^ A further limitation of the UK Biobank analyses is that, like smoking, all covariates’ data were only available at baseline. Moreover, there are no data on AAA size, since neither UK Biobank nor the sources used to ascertain AAA incidence hold data on aortic diameter. Additionally, as discussed, the RR estimates for smoking and AAA may be influenced by the denominator effect, where the low absolute risk in female never smokers amplifies the RR, even if the absolute difference remains modest. This highlights the importance of considering both absolute and relative measures when interpreting sex-specific differences in risk, which these data provide.^32^

In conclusion, the data here confirm that smoking is a much stronger RR factor for AAA in women than in men, perhaps underpinned by the observations that AAA is rare in women who have never smoked and that women with incident AAA may smoke more cigarettes per day than men with incident AAA. Within this study, there was little evidence to support the hypothesis that inflammation mediated the sex disparity, and further research is needed. These observations should emphasize the need to both improve smoking cessation efforts in women and men and, particularly, to reevaluate AAA screening for women smokers.

## Article Information

### Acknowledgments

This study would not have been possible without the participants, investigators, and funders of UK Biobank. The authors thank Dr Frederick Ho, University of Glasgow, for advice on statistical analyses.

### Sources of Funding

None.

### Disclosures

None.

### Supplemental Material

Tables S1–S3

Figures S1–S6

Major Resources Table
